# A study of dealing class imbalance problem with machine learning methods for code smell severity detection using PCA-based feature selection technique

**DOI:** 10.1038/s41598-023-43380-8

**Published:** 2023-09-27

**Authors:** Rajwant Singh Rao, Seema Dewangan, Alok Mishra, Manjari Gupta

**Affiliations:** 1 Department of Computer Science and Information Technology, Guru Ghasidas Vishwavidyalaya, Bilaspur, India; 2 Faculty of Engineering, Norwegian University of Science and Technology, Trondheim, Norway; 3 (Computer Science), DST - Centre for Interdisciplinary Mathematical Sciences, Institute of Science, Banaras Hindu University, Varanasi, India

**Keywords:** Engineering, Mathematics and computing

## Abstract

Detecting code smells may be highly helpful for reducing maintenance costs and raising source code quality. Code smells facilitate developers or researchers to understand several types of design flaws. Code smells with high severity can cause significant problems for the software and may cause challenges for the system's maintainability. It is quite essential to assess the severity of the code smells detected in software, as it prioritizes refactoring efforts. The class imbalance problem also further enhances the difficulties in code smell severity detection. In this study, four code smell severity datasets (Data class, God class, Feature envy, and Long method) are selected to detect code smell severity. In this work, an effort is made to address the issue of class imbalance, for which, the Synthetic Minority Oversampling Technique (SMOTE) class balancing technique is applied. Each dataset's relevant features are chosen using a feature selection technique based on principal component analysis. The severity of code smells is determined using five machine learning techniques: K-nearest neighbor, Random forest, Decision tree, Multi-layer Perceptron, and Logistic Regression. This study obtained the 0.99 severity accuracy score with the Random forest and Decision tree approach with the Long method code smell. The model's performance is compared based on its accuracy and three other performance measurements (Precision, Recall, and F-measure) to estimate severity classification models. The impact of performance is also compared and presented with and without applying SMOTE. The results obtained in the study are promising and can be beneficial for paving the way for further studies in this area.

## Introduction

The proper and efficient maintenance of software has always been a challenge for the industry, researchers, or software professionals. The maintenance becomes even more challenging if the software developed is complex one. And nowadays, software's complexity is rising due to the increased module numbers and their size, complicated requirements, and also due to the significant presence of code smells in the developed software. The complexities are challenging to evaluate, comprehend, and go beyond developers' scope, posing obstacles in development as well as in software maintenance. However, researchers have found methods to avoid complexities in the developmental stage and hence ultimately, ease the maintenance efforts. One such method is identifying code smells and fixing them to simplify the software's interface, precise, uncomplicated to create and maintain^1^. Developers must follow the required software quality standards by using functional and nonfunctional concepts in the software improvement process^2^. It has been quite evident that developers focus only on functional needs while ignoring nonfunctional needs, including maintainability, credibility, reprocessability, and accessibility^3^. The lack of focus on nonfunctional requirements reduces software quality and ultimately increases the software maintenance effort and difficulties. The method of software quality assurance includes software inspection as a fundamental component^4^. The quality of software is heavily influenced by the quality of the process employed during its development. The software process can be characterized, controlled, evaluated, and enhanced^5^.

The code smell severity (CSS) is another significant factor to consider when evaluating the success of code smell detection is important because it lets refactoring actions be put in order of importance. CSS describes the degree of code smells that may occur during software development and maintenance. High-severity code smells might develop into a serious and complicated issue for the software's maintenance procedure. Another element of code smell detection which did not acquire much consideration in the research was the notion that distinct code smell occurrences might vary in size, strength, or severity and should, as a result, be handled differently due to their differing effects on the quality of software. This study outlined the essential elements of machine learning (ML) models. We focused on the possibility of classifying the existence or nonexistence of code smells and the severity of such smells. Every code smell is bad for the software quality in its own way. When determining the CSS, we estimate how many of these qualities are present. For instance, if a God Class is extremely massive, complicated, or concentrates a significant proportion of the system's intellect, it has high severity^6^.

In our previous studies^7–10^, and^11^ we applied binary classification (to classify the code smell’s existence or nonexistence) to four datasets of code smell [God class (GC), Data class (DC), Feature envy (FE), and Long method (LM)], and got favorable results. The purpose of this research study is to assess the effectiveness of classifying code smells according to their severity and to determine the most effective method in this regard. A dataset containing 420 data samples (classes or methods) and code smells taken from 76 open-source Java projects is used to evaluate the collection of techniques. Good performance in rating the results of code smell detection is essential for providing software developers with trustworthy information for prioritizing their refactoring and reengineering efforts, for example, suggesting them to repair only the smells with maximum severity.

To the best of our knowledge, the CSS classification on the same dataset was worked by Fontana et al.^6^ and Abdou et al.^12^. We have observed some limitations of these as follows:

1. The dataset suffers from an issue of class imbalance, which has not been addressed.
2. Class based accuracy was not illustrated.
3. Performance measurements like Recall, F-measure, and Precision was not given.

The followings are our empirical investigation; we identified following strong motivational research queries (RQ) concerning the need for CSS detection:

- *RQ1* Which ML algorithm is the most effective for detecting severities of code smell?
- *Motivation *Fontana et al.^6^ and Alazba et al.^13^ applied various ML algorithms and compared the performances of ML algorithms. Therefore, we applied five ML algorithms to investigate and observe the performance and find the best algorithm for CSS detection.
- *RQ2* What is the impact of the class balancing method (SMOTE) on the performance of various models on the CSS detection?
- *Motivation* To address the issue of class imbalance, Pandey et al.^14^ applied a random sampling technique. Therefore, we used the SMOTE method to determine how the class imbalance issue affected the level of code smell detection.
- *RQ3* What is the impact of feature selection technique (FST) on the performance of various models on the CSS detection?
- *Motivation* Dewangan et al.^7^, and Mhawish et al.^15,16^, investigated the effect of several FSTs on performance measures. They discovered that using FST improved performance accuracy. So, to examine the impact of the FST on improving the method's accuracy and extracting code smell severities, which contributes a substantial role in the CSS detection process.

To address the above three research questions, our key contributions to this study are as follows:

1. This study addresses the class imbalance problem and applies SMOTE class balancing technique to the four CSS datasets.
2. A principal component analysis (PCA)-based FST is used to show the result of FST on the model performance for detecting the severity of code smells.
3. We have applied five ML models: Logistic Regression (LR), Multi-layer Perceptron (MLP), Random forest (RF), Decision tree (DT), and K-nearest neighbour (KNN).
4. We have considered four performance measurements: Precision, Recall, F-measure, and Severity Accuracy Score for each severity class for the severity dataset of each code smell.

Thus, our study applied the SMOTE method to handle the class imbalance issue and the Principal Component Analysis (PCA)-based feature selection technique to improve the model accuracy and achieved a severity accuracy score of 0.99 using the Random Forest and Decision Tree algorithms in the context of detecting the Long Method code smell.

The paper is organized as follows: "Background/literature review" section discusses related works and provides a brief description of CSS detection by applying ML algorithms. “Description of the proposed model and dataset” section discusses the dataset's description and proposed models, the experimental results of the proposed model are described in "Experiment results" and "Discussion and result analysis" section outlines the discussion and compares our outcomes with other related studies, and finally the last "Conclusion" section concludes with future research directions.

## Background/literature review

Various research studies^6,17–19^ on CSS detection and how it affects the model performance have been conducted. Many techniques (i.e., machine learning, ensemble learning, and class imbalance problem) are presented in the literature to identify the severity of code smells. Each technique yields a unique set of outcomes. In this section, we have presented the related work by dividing it into three sub-sections. The first discusses the ML-based, the second ensemble learning-based, and the third reviews the class imbalance problem for CSS detection.

### Code smell severity (CSS) detection based on the machine learning algorithms

Numerous researchers have employed a variety of machine learning (ML) algorithms to detect CSS. Fontana et al.^6^ explored a range of ML techniques, including regression, multinomial classification, and a binary classifier for ordinal classification. Their evaluation demonstrated a correlation between predicted and actual severity, achieving 88–96% accuracy measured by Spearman's p. Another study by Abdou et al.^12^ utilized different ML models, including ordinal, regression, and multinomial classifiers for CSS classification. They also applied the LIME approach to interpret ML models and rules of prediction, utilizing the PART algorithm to assess feature efficiency. The highest accuracy they achieved was 92–97% using the Spearman algorithm correlation measurement.

Tiwari et al.^17^ introduced a tool to identify long methods and their severity, emphasizing the significance of refactoring long methods. Their findings showed that this tool matched expert evaluations for approximately half of the approaches with a one-level tolerance. Additionally, they identified high severity evaluations that closely aligned with expert judgments.

For closed-source software bug reports with varying degrees of severity, Baarah et al.^18^ investigated the use of eight ML models, including Support Vector Machine, Naive Bayes, Naive Bayes Multinomial, Decision Rules (JRip), Decision Tree (J48), Logistic Model Trees, K-Nearest Neighbor, and Random Forest. The Decision Tree (DT) model outperformed the others with 86.31% accuracy, 90% Area under the Curve (AUC), and 91% F-measure.

Gupta et al.^19^ introduced a hybrid technique to assess code smell intensity in the Kotlin language and identified identical code smells in the Java language. Their work involved applying various ML models, with the JRip algorithm achieving the best outcome at 96% precision and 97% accuracy.

Hejres et al.^20^ utilized three ML models (J48, SMO, and ANN) to detect CSS from four datasets. The SMO model yielded the best results for the god class and feature envy datasets, while the ANNE with the SMO model showed the highest accuracy for the long method dataset.

In their study, Hu et al.^21^ reexamine the efficacy of ten classification approaches and eleven regression methods for predicting code severity. The evaluation of these methods is based on two key performance metrics: the Cumulative Lift Chart (CLC) and Severity@20%. Additionally, Accuracy is considered as a secondary performance indicator. The findings indicate that the Gradient Boosting Regression (GBR) technique has superior performance in relation to these criteria.

Sandouka et al.^22^ proposed a Large Class and Long Method code smell based Python code smell dataset. They utilized six ML models for Python code smell detection. They measure the Accuracy and MCC percentage. They obtained the 0.89 best MCC rate using the DT model.

Zakeri-Nasrabadi et al.^23^ surveyed 45 pre-existing datasets to examine the factors contributing to a dataset's effectiveness in detecting smells. They found that the suitability of a dataset for this purpose is heavily influenced by various properties, including its size, severity level, project types, number of each type of smell, overall number of smells, and the proportion of smelly to non-smelly samples within the dataset. Most currently available datasets support identifying code smells such as God Class, Long Method, and Feature Envy. However, it is worth noting that there are six code smells included in Fowler and Beck's catalog that do not have corresponding datasets available for analysis. It may be inferred that the current datasets exhibit imbalanced sample distributions, a shortage of severity level support, and a limitation to the Java programming language.

### Code smell severity (CSS) detection based on the ensemble and deep learning algorithms

Numerous research studies have explored the application of various ensemble learning methods for code smell detection. Alazba et al.^13^ conducted experiments with fourteen ML and stacking ensemble learning methods with six datasets for code smells and reported a remarkable accuracy of 99.24% with LM Dataset using the Stack-SVM algorithm.

Malathi et al.^24^ introduced a deep learning approach for detecting class code smells. This approach leverages a diverse set of characteristics specifically designed for different types of code smells. This deep learning model would effectively detect instances belonging to the single class CS only. Therefore, this paper proposes an advanced Deep Learning Based many Class type Code Smell detection (DLMCCMD) to automatically detect many kinds of Code Smells, such as huge class, misplaced class, lazy class, and data clumps. The CNN-LSTM architecture has been devised for the purpose of classifying a certain feature that encompasses both source code information and code metrics. The acquired data is consolidated to conduct positive testing of source code programs with reduced computational time.

Dewangan et al.^25^ utilized four ML (LR, RF, KNN, DT) and three ensemble models (AdaBoost, XG Boost, and Gradient Boosting) to detect CSS from four datasets. They used chi-square FST and two-parameter optimization methods (Grid search and Random search). They obtained that the XG Boost model achieved a high accuracy rate of 99.12% when applied to the Long method code smell dataset, utilizing the Chi-square-based feature selection strategy.

Nanda et al.^26^ employed a hybrid approach that integrated the Synthetic Minority Over-sampling Technique (SMOTE) with the Stacking model to effectively classify datasets related to the severity of DC, GC, LM, and FE, achieving performance improvement from 76 to 92%.

Pushpalatha et al.^27^ proposed a method for predicting bug report severity in closed-source datasets, utilizing the NASA project dataset (PITS) from the PROMISE Repository. To enhance accuracy, they employed ensemble learning methods and two-dimensional reduction techniques, including information gain and chi-square.

Zhang et al.^28^ introduced MARS, a brain-inspired method for code smell detection that relies on the Metric-Attention method. They applied various ML and Deep learning models and found that MARS outperformed conventional techniques in terms of accuracy.

Liu et al.^29^ presented a severity prediction approach for bug reports based on FSTs and established a ranking-based policy to enhance existing FSTs and create an ensemble learning FST by combining them. Among the eight FST methods applied, the ranking-based approach achieved the highest F1 score of 54.76%.

Abdou et al.^30^ suggested using ensemble learning techniques to detect software defects. They explored three ensemble approaches: Bagging, Boosting, and Rotation Forest, which combine re-sampling techniques. The experiments conducted on seven datasets from the PROMISE repository showed that the ensemble method outperforms single learning methods, with the rotation forest using the re-sampling approach achieving a maximum accuracy of 93.40% for the KC1 dataset.

Dewangan et al.^11^ employed ensemble and deep learning methods to discover code smells. They achieved a remarkable 100% accuracy for the LM dataset by applying all ensemble methods and using Chi-square FST and SMOTE class balancing methods.

### Code smell severity (CSS) detection dealing with class imbalance problem

Zhang et al.^31^ proposed a DeleSmell method to identify the code smells using a deep learning model. They constructed the dataset by collecting data from 24 real-world projects. To address the unbalance in the dataset, a refactoring technique is intended to automatically change useful source code into smelly code and to generate positive data using actual cases. They employed the SVM method and found that DeleSmell enhances the efficiency of brain class code smell detection by up to 4.41% compared to conventional techniques. Pecorelli et al.^32^ implemented five imbalance techniques (Class Balancer, SMOTE, Resample, and Cost-Sensitive Classifier, One Class Classifier) to identify the impact of five code smell detection on the various ML algorithms. They found that ML models relying on SMOTE obtained the best performance. A random sampling approach was applied by Pandey et al.^14^ to address the problem of class imbalance. With the random sampling technique, they discovered better results.

The related work summarizes that various authors used machine learning techniques (machine learning, ensemble learning, and deep learning). “Code smell severity (CSS) detection based on the machine learning algorithms”, “Code smell severity (CSS) detection based on the ensemble and deep learning algorithms”, and “Code smell severity (CSS) detection dealing with class imbalance problem” sections discussed all related studies which worked on the CSS datasets. The above literature has some limitations, only some studies have solved the class imbalance problem in the datasets, but they need to address the dataset's class-wise accuracy. Also, only some studies have used the feature selection technique and examined its effect on performance accuracy.

## Description of the proposed model and dataset

**Figure 1:**
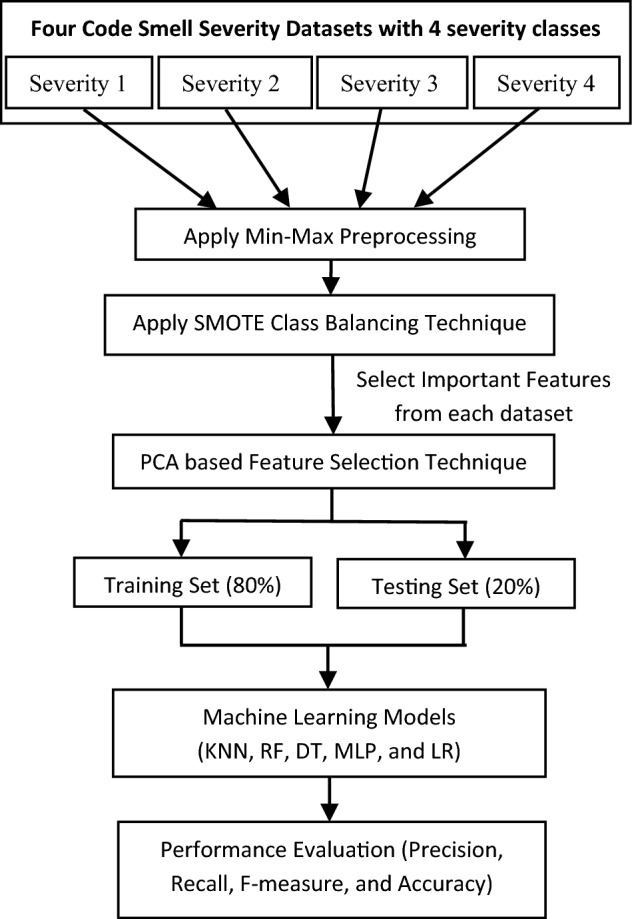
Proposed model.

We followed the following steps to detect the severity, as depicted in Fig. 1. Fontana et al.^6^ served as the source for initially deriving four datasets on CSS. The min–max preprocessing technique was used to ensure data comparability, normalizing data values to fall within the range of 0–1. A SMOTE class balancing algorithm is applied to handle the class imbalance issues. Next, a PCA-based FST technique was used to select the most relevant features from each dataset. Subsequently, the dataset was into two parts: an 80% training set for model training and a separate test set for model evaluation (fivefold cross validation). Finally, machine learning algorithms were applied, and performance evaluations were conducted. The entire procedure conducted in this study is outlined in Fig. 1.

### Description of the dataset

The four datasets from Fontana et al.^6^ that are being considered are divided into two class-level datasets (DC, GC) and two method-level datasets (FE, LM). Visit http://essere.disco.unimib.it/reverse/MLCSD.html to access each of these datasets. Out of 111 systems, 76 have been selected by Fontana et al.^6^ and have been computed using a variety of sizes and a significant amount of object-oriented features. For the system selection, they considered the systems Qualitas Corpus compiled by Tempero et al.^33^. These methods included iPlasma (Brain Class, GC), Anti-pattern Scanner^34^, PMD^35^, iPlasma, Fluid Tool^36^, and Marinescu detection rules^37^ for determining the intensity of code smells. Table 1 displays the automatic detection tools.

**Table 1:** Automatic detector tools (advisors)^6^.

Code smell	Reference, tool/detection rules
DC	iPlasma, anti-pattern scanner^34^, fluid tool^36^,
GC	iPlasma (GC, brain class), PMD^35^
FE	iPlasma, fluid tool^36^
LM	iPlasma (brain method), PMD, Marinescu detection rule^37^

### Code smells severity classification

After manually assessing each instance of a code smell, a severity score is assigned.

- 1: A class or method that is unaffected receives a score of 1 for "No smell”;
- 2: A class or function that is only marginally affected receives a score of 2 for a non-severe smell;
- 3 : A class or method receives a smell score of 3 if it possesses all of the qualities of a smell;
- 4: There is a severe smell, and its size, complexity, and coupling values are extremely high. It receives a score of 4.

The datasets are defined below:

- *DC* It refers to classes that hold fundamental data with essential functionality and are extensively utilized by other classes. A DC typically exposes numerous features through simple accessor methods, presenting a straightforward and uncomplicated design^6^.
- *GC* It refers to classes that centralize the system's intelligence, often being considered one of the most complex code smells. GCs tend to accumulate numerous responsibilities, actions, and tasks, leading to issues related to code size, coupling, and complexity^6^.
- *FE* It pertains to techniques or methods that heavily rely on data from classes other than their own. It shows a preference for utilizing features exposed through accessor methods in other classes^6^.
- *LM* It describes strategies or procedures that concentrate a class's functionality, frequently leading to long and complicated code. Because they rely so largely on information from other classes, LMs are difficult to understand^6^.

### Dataset structure

Each dataset contains 420 instances (classes or methods). Specifically, 63 instances are selected for the DC and GC datasets, while 84 instances are chosen for the FE and LM datasets. The dataset configuration, as shown in Table 2, includes the distribution of instances across severity levels. It is observed that severity level 2 has the least number of occurrences in the datasets. Additionally, the class-based smells (DC and GC) exhibit a different balance of severity levels 1 and 4 compared to the method-based smells (FE and LM)^6^.

**Table 2:** Dataset configuration^6^.

CSS datasets	Severity			
	1	2	3	4
DC	151	32	113	124
GC	154	29	110	127
FE	280	23	95	22
LM	280	11	95	34

### Preprocessing technique

The datasets encompass a diverse set of features; consequently, it is preferable to normalize the features before using the ML techniques. In this study, the Min–Max preprocessing method is used to rescale datasets with feature or observation values ranging from 0 to 1^38^. The min–max formula, as presented in Eq. 1, calculates the normalized value denoted by X', based on the original real value represented by X. The feature's minimum value (Xmin) is set to "0," and the maximum value (Xmax) is set to "1." All other values are scaled proportionally as decimals within the range of 0–1.

1 $$ X^{\prime} = \frac{X - X\min }{{X\max - X\min }} $$

### Class balancing technique

From Table 2, we observed that the dataset (Fontana et al.^6^) has four types of severity levels (metrics). The distribution of each severity level of each dataset is different. The class distribution of this dataset is not balanced. In this research, each class of each dataset was balanced using the SMOTE class balancing approach. SMOTE is a well-known oversampling method that was developed to improve random oversampling^39^.

### Feature selection technique

Feature selection aims to identify the most relevant features in a dataset, enhancing model performance by better understanding the instances that contribute to distinguishing parallel roles in features^40^. In this study, we utilized the PCA (Principal Component Analysis) feature selection technique to extract the most informative features from each dataset. PCA is a dimensionality-reduction method commonly employed to reduce the number of variables in large datasets, creating a smaller set that preserves most of the data's variability^41^. The discussion of the selected best features/instances from each dataset and their impact on performance accuracy is provided in “Effect of PCA feature selection technique on the model’s severity accuracy score” section.

### Machine learning models

Machine learning is a computational approach that encompasses a range of methodologies employed by computers to make predictions, enhance predictive accuracy, and forecast behavior patterns using datasets^42^. In this study, we have applied five ML models to detect the CSS from CSS datasets. The five ML models are Logistic regression, Multi-layer perceptron, Random forest, Decision tree, and K-nearest neighbor. The five ML modes described in following subsections:

#### Logistic regression (LR)

To analyze and categorize binary and proportional response data sets, researchers frequently use the LR method, one of the most significant statistical and data mining approaches. One of its key features is that LR may extend to multi-class classification problems and automatically generate probability.

#### Multi-layer perceptron (MLP)

This classifier is made up of layers of units. Each node in the fully linked network under consideration here comprises a layer. In that layer, every other node is connected to every other node in the layer below it. A minimum of three layers, including an input layer, one or more hidden layers, and an output layer, make up each MLP. The input layer divides up the inputs among the following levels. Input nodes lack thresholds and have linear activation functions. There are thresholds connected to the minimum addition to the weights for each hidden unit node and each output node. The outputs have linear activation functions, while the buried unit nodes have nonlinear activation functions.

#### Random forest (RF)

In the proposed model, we employed Random Forest (RF) as the machine learning classifier. In RF, each tree depends on the values of a random vector sampled randomly, and this sampling is done with the same distribution for all the trees in the forest. With an increasing number of trees in the forest, the generalization error asymptotically converges to a limit. The overall generalization error of the forest of tree classifiers is determined by the quality of each individual tree and the relationships between them.

#### Decision tree (DT)

In a DT model, each internal branch is connected to a decision, and the leaf node is often connected to a result or class label. Each internal node tests one or more attribute values that result in two or more links or branches. Each connection has a potential decision value attached to it. These connections are distinct and comprehensive^7^.

#### K-nearest neighbor (KNN)

The KNN method is a supervised ML technique used for classification prediction issues. Meanwhile, most of its applications in the industry are for classification prediction issues. The KNN model uses "feature similarity" to predict the value of a new data point, which also implies that the value will depend on how closely the new data point resembles the training point^7^.

### Performance evaluations

**Figure 2:**
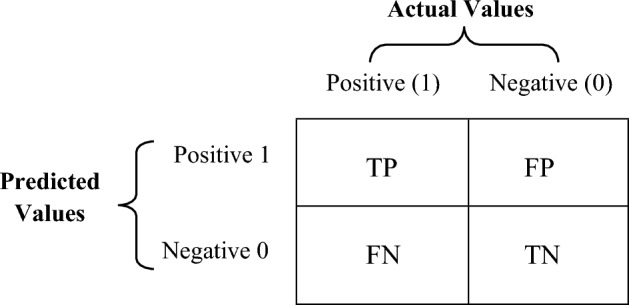
Confusion matrix^44^.

We employed four performance evaluations—Precision, Recall, F-measure, and Severity Accuracy Score to determine the performance of five machine learning models. These evaluation indicators are described briefly in following subsections. Four terms are considered while calculating the performance evaluation: True positive (TP), False positive (FP), True negative (TN), and False negative (FN). The confusion matrix (CM) calculates these four terms, which contains the actual and predicted values recognized by CSS models. Figure 2 shows the confusion matrix prediction.

#### Precision (P)

Precision (P) is concerned with the accurate identification of code smell severities by the ML model^43^. To calculate precision, Eq. (2) is employed, where precision is determined by dividing the number of true positives (TP) by the sum of TP and false positives (FP).

2 $$ Precision\, \left( P \right) = \frac{TP}{{TP + FP }} $$

#### Recall (R)

Recall (R) pertains to the accurate identification of code smell severities by the ML model^43^. To calculate recall, we use Eq. (3), which involves dividing the number of true positives (TP) by the sum of TP and false negatives (FN).

3 $$ Recall\, \left( R \right) = \frac{TP}{{TP + FN}} $$

#### F-measure (F)

F-measure (F) deals with the harmonic mean of precision and recall, and it's set for a balance between their values^43^. Its value lies between 0 and 1, 0 is the poorest performance and 1 is the most excellent performance. Equation (4) is applied to calculate F-measure.

4 $$ F - measure\,\left( F \right) = 2 \times \frac{precision \times recall}{{precision + recall}} $$

#### Severity accuracy score (SAS)

Severity accuracy score (SAS) deals with the organization of precision and recall. It illustrates the measurement of exactly classified instances in the positive and negative classes^43^. Equation (5) is used to compute accuracy. SAS is considered as dividing the sum of the TP and TN by the sum of the TP, TN, FP, and FN.

5 $$ {\text{Severity Accuracy Score }}\left( {{\text{SAS}}} \right) = \frac{TP + TN}{{TP + TN + FP + FN}} $$

## Experiment results

To address RQ1, five ML models are used. The datasets GC, DC, FE, and LM for the severity of code smells are chosen. In this study, each dataset has four categories of severity (severity 1, severity 2, severity 3, and severity 4). We have shown individual outcomes for each dataset's severity level. In addition, the average outcome of all severity classifications is also presented. The following “Outcomes for data class” to “Outcomes for long method” sections, display the experimental outcomes of five ML models with fivefold cross validation: LR, MLP, RF, DT, and KNN, in tabular form for four datasets.

### Outcomes for data class

**Figure 3:**
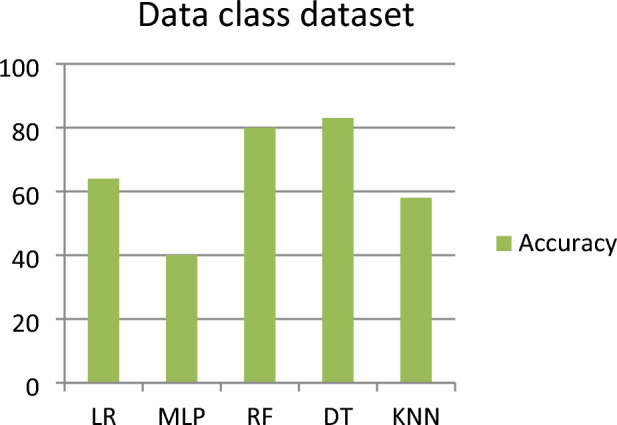
Accuracy comparison of data class dataset for all the classifier.

This subsection represents the effect of applying the five ML models to the DC dataset. Table 3 shows the severity detection outcomes with four measurements (Precision, Recall, F-measure, and Severity Accuracy Score) for the DC dataset (for each level of severity, with the average of all levels of severity) applying five ML models. Figure 3 shows the accuracy comparison of the data class dataset for all the classifiers. For the DC dataset, it has been observed that the DT model detected the highest severity accuracy score (with an average of all the severity classes) of 0.83, the precision of 0.84, recall of 0.83, and F-measure of 0.84, while the worst severity of 0.40 accuracy was detected by the MLP model.

**Table 3:** Outcomes for data class dataset.

Model name	Severity classes	Precision	Recall	F-measure	Severity accuracy score
LR	Severity 1	0.77	0.89	0.83	0.89
	Severity 2	0.51	0.54	0.53	0.54
	Severity 3	0.49	0.49	0.49	0.49
	Severity 4	0.81	0.64	0.71	0.64
	Average of all severity class	0.65	0.64	0.64	0.64
MLP	Severity 1	0.81	0.39	0.53	0.58
	Severity 2	0.27	0.92	0.42	0.92
	Severity 3	0.33	0.03	0.6	0.74
	Severity 4	0.67	0.38	0.48	0.59
	Average of all severity class	0.54	0.40	0.37	0.40
RF	Severity 1	0.97	0.88	0.92	0.88
	Severity 2	0.83	1.00	0.91	1.00
	Severity 3	0.59	0.71	0.65	0.71
	Severity 4	0.88	0.66	0.75	0.66
	Average of all severity class	0.82	0.80	0.80	0.80
DT	Severity 1	0.97	0.95	0.94	0.95
	Severity 2	0.85	0.92	0.88	0.92
	Severity 3	0.70	0.74	0.72	0.74
	Severity 4	0.83	0.75	0.79	0.75
	Average of all severity class	**0.84**	**0.83**	**0.84**	**0.83**
KNN	Severity 1	0.68	0.54	0.60	0.54
	Severity 2	0.65	0.62	0.63	0.62
	Severity 3	0.45	0.71	0.56	0.71
	Severity 4	0.62	0.48	0.54	0.48
	Average of all severity class	0.61	0.58	0.58	0.58

Significant values are in bold.

### Outcomes for god class

**Figure 4:**
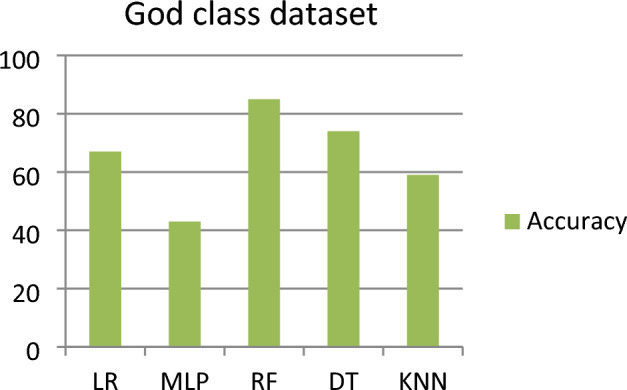
Accuracy comparison of god class dataset for all the classifier.

This subsection represents the effect of applying the five ML models to the GC dataset. Table 4 shows the severity detection outcomes with four measurements for the GC dataset (for each level of severity, with the average of all levels of severity) applying five ML models. Figure 4 shows the accuracy comparison of the god class dataset for all the classifiers. For the GC dataset, it has been observed that the RF model detected (with an average of all the severity classes) the highest Severity Accuracy Score, precision, recall, and F-measure of 0.85, while the worst Severity Accuracy Score is 0.43 was detected by the MLP model.

**Table 4:** Outcomes for god class dataset.

Model name	Severity classes	Precision	Recall	F-measure	Severity accuracy score
LR	Severity 1	0.80	0.90	0.84	0.89
	Severity 2	0.69	0.56	0.62	0.56
	Severity 3	0.51	0.53	0.52	0.53
	Severity 4	0.68	0.69	0.68	0.69
	Average of all severity class	0.67	0.67	0.67	0.67
MLP	Severity 1	0.43	0.74	0.55	0.74
	Severity 2	0.64	0.32	0.42	0.41
	Severity 3	0.25	0.24	0.24	0.56
	Severity 4	0.58	0.37	0.45	0.37
	Average of all severity class	0.46	0.43	0.42	0.43
RF	Severity 1	0.97	0.83	0.89	0.83
	Severity 2	0.82	0.96	0.89	0.96
	Severity 3	0.83	0.77	0.80	0.77
	Severity 4	0.78	0.83	0.81	0.83
	Average of all severity class	**0.85**	**0.85**	**0.85**	**0.85**
DT	Severity 1	0.89	0.71	0.79	0.71
	Severity 2	0.77	0.96	0.86	0.96
	Severity 3	0.64	0.58	0.61	0.58
	Severity 4	0.67	0.73	0.70	0.73
	Average of all severity class	0.75	0.74	0.74	0.74
KNN	Severity 1	0.59	0.63	0.61	0.63
	Severity 2	0.73	0.79	0.76	0.78
	Severity 3	0.44	0.61	0.51	0.62
	Severity 4	0.71	0.33	0.45	0.33
	Average of all severity class	0.62	0.59	0.58	0.59

Significant values are in bold.

### Outcomes for feature envy

**Figure 5:**
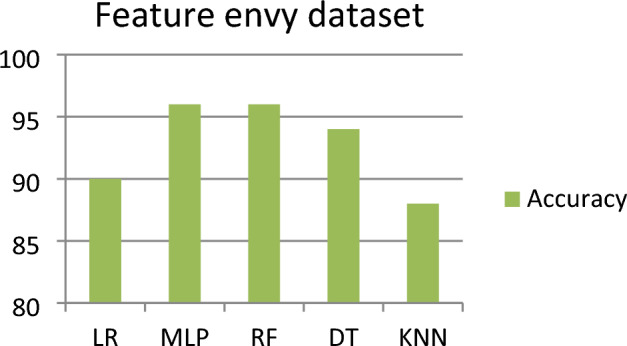
Accuracy comparison of feature envy dataset for all the classifier.

This subsection represents the effect of applying the five ML models to the feature envy dataset. Table 5 shows the severity detection outcomes with four measurements for the feature envy dataset (for each level of severity, with the average of all levels of severity) applying five ML models. Figure 5 shows the accuracy comparison of the feature envy dataset for all the classifiers. For the feature envy dataset, it has been observed that the MLP and RF model detected (with an average of all the severity classes) the highest Severity Accuracy Score, precision, and recall of 0.96 and the F-measure is 0.96 for the RF model and 0.95 for MLP. The worst Severity Accuracy Score is 0.90, detected by the LR model.

**Table 5:** Outcomes for feature envy dataset.

Model name	Severity classes	Precision	Recall	F-measure	Severity accuracy score
LR	Severity 1	0.96	0.92	0.94	0.91
	Severity 2	0.90	0.90	0.90	0.90
	Severity 3	0.83	0.79	0.81	0.79
	Severity 4	0.93	1.00	0.96	1.00
	Average of all severity class	0.90	0.90	0.90	0.90
MLP	Severity 1	1.00	0.96	0.92	0.96
	Severity 2	0.95	0.98	0.97	0.98
	Severity 3	0.91	0.96	0.93	0.96
	Severity 4	0.97	1.00	0.98	1.00
	Average of all severity class	**0.96**	**0.96**	**0.95**	**0.96**
RF	Severity 1	1.00	0.88	0.94	0.88
	Severity 2	0.90	1.00	0.95	1.00
	Severity 3	0.94	0.94	0.94	0.94
	Severity 4	1.0	0.98	0.99	0.98
	Average of all severity class	**0.96**	**0.96**	**0.96**	**0.96**
DT	Severity 1	1.00	0.92	0.96	0.92
	Severity 2	0.95	0.95	0.95	0.95
	Severity 3	0.83	0.96	0.89	0.96
	Severity 4	1.00	0.93	0.97	0.93
	Average of all severity class	0.95	0.94	0.94	0.94
KNN	Severity 1	1.00	0.72	0.84	0.72
	Severity 2	0.85	0.98	0.91	0.98
	Severity 3	0.85	0.77	0.81	0.77
	Severity 4	0.86	0.98	0.92	0.98
	Average of all severity class	0.88	0.88	0.87	0.88

Significant values are in bold.

### Outcomes for long method

**Figure 6:**
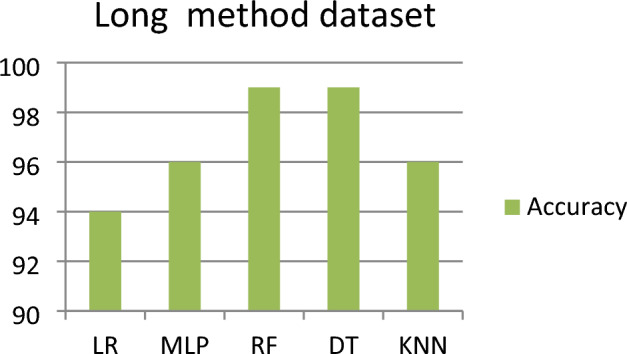
Accuracy comparison of long method dataset for all the classifier.

This subsection represents the effect of applying the five ML models to the LM dataset. Table 6 shows the severity detection outcomes with four measurements for the LM dataset (for each level of severity, with the average of all levels of severity) applying five ML models. Figure 6 shows the accuracy comparison of the long method dataset for all the classifiers. For the LM dataset, we observed that the RF and DT both models detected (with an average of all the severity classes) the highest Severity Accuracy Score, precision, recall, and F-measure of 0.99, while the worst Severity Accuracy Score is 0.94 detected by the LR model.

**Table 6:** Outcomes for long method dataset.

Model Name	Severity classes	Precision	Recall	F-measure	Severity accuracy score
LR	Severity 1	1.00	0.97	0.98	0.97
	Severity 2	0.94	1.00	0.97	1.00
	Severity 3	0.92	0.85	0.88	0.85
	Severity 4	0.91	0.93	0.91	0.93
	Average of all severity class	0.94	0.94	0.94	0.94
MLP	Severity 1	0.96	1.00	0.98	1.00
	Severity 2	0.97	0.97	0.97	0.97
	Severity 3	0.96	0.88	0.92	0.88
	Severity 4	0.96	1.00	0.98	1.00
	Average of all severity class	0.96	0.96	0.96	0.96
RF	Severity 1	1.00	0.99	0.99	0.99
	Severity 2	1.00	1.00	1.00	1.00
	Severity 3	0.97	1.00	0.98	1.00
	Severity 4	1.00	0.98	0.99	0.98
	Average of all severity class	**0.99**	**0.99**	**0.99**	**0.99**
DT	Severity 1	1.00	0.99	0.99	0.99
	Severity 2	1.00	1.00	1.00	1.00
	Severity 3	0.97	0.99	0.98	0.99
	Severity 4	0.99	0.99	0.99	0.99
	Average of all severity class	**0.99**	**0.99**	**0.99**	**0.99**
KNN	Severity 1	1.00	0.96	0.98	0.96
	Severity 2	0.91	0.98	0.95	0.98
	Severity 3	0.96	0.91	0.93	0.91
	Severity 4	0.98	0.98	0.98	0.98
	Average of ALL SEVERITY CLASS	0.96	0.96	0.96	0.96

Significant values are in bold.

### The impact of SMOTE's class-balancing method on predictive performance

RQ2 was addressed using the SMOTE class balancing method. This experiment is done to observe SMOTE's impact on balancing the classes of four severity code smell datasets. Table 7 shows how each model's performance Severity Accuracy Score gets affected for four CSS datasets. According to the comparison, the SMOTE class balancing methodology helps almost all ML techniques improve their Severity Accuracy Score for all datasets, and it affects each model and each dataset in slightly different ways.

**Table 7:** Result Comparison between with and without applied SMOTE.

Dataset	Model name	Severity accuracy score with applied SMOTE	Severity accuracy score without applied SMOTE
DC	LR	0.64	**0.73**
	MLP	**0.40**	0.39
	RF	**0.80**	0.79
	DT	**0.83**	0.70
	KNN	**0.58**	0.48
GC	LR	0.67	**0.68**
	MLP	0.43	0.43
	RF	**0.85**	0.76
	DT	**0.74**	0.70
	KNN	**0.59**	0.40
FE	LR	**0.90**	0.89
	MLP	**0.96**	0.79
	RF	**0.96**	0.89
	DT	**0.94**	0.89
	KNN	**0.88**	0.65
LM	LR	**0.94**	0.91
	MLP	**0.96**	0.89
	RF	**0.99**	0.98
	DT	**0.99**	0.97
	KNN	**0.96**	0.94

Significant values are in bold.

We observed the following points for each dataset:

1. For the DC dataset, MLP, RF, DT, and KNN models provided higher Severity Accuracy Scores when we used SMOTE technique, while the LR model achieved a better Severity Accuracy Score without using SMOTE technique. The DT model achieved the highest Severity Accuracy Score of 0.83 using the SMOTE balancing technique.
2. For the GC dataset, RF, DT, and KNN models provided higher Severity Accuracy Scores when we applied SMOTE technique, while the LR model achieved a better Severity Accuracy Score without using SMOTE technique, and the MLP model presented the same results for both with and without applied SMOTE balancing technique. The RF model achieved the highest Severity Accuracy Score of 0.85 using the SMOTE balancing technique.
3. For the FE dataset, all five models presented higher Severity Accuracy Score when we applied SMOTE technique. The highest Severity Accuracy Score of 0.96 was achieved by the MLP and RF model using SMOTE balancing technique.
4. For the LM dataset, all five models provided higher Severity Accuracy Score when we SMOTE technique. The RF and DT model obtained the highest Severity Accuracy Score of 0.99 using the SMOTE balancing technique.

### Effect of PCA feature selection technique on the model’s severity accuracy score

In this study, we have applied the PCA-based FST to select the best features from the severity dataset. The PCA selects the DC dataset with eight components, the GC dataset with nine components, the FE dataset with nine components, and the LM dataset with ten components. Table 8 shows the best-selected features from each dataset using PCA. All selected feature descriptions are provided in the appendix section of Table 12.

**Table 8:** PCA-selected features from each dataset.

Dataset	No. of components	Components selected by PCA
DC	08	NOMNAMM_project, LOC_project, LOCNAMM_type, LOC_package, LOC_type, NOCS_project, NOCS_package, NOMNAMM_package
GC	09	NOMNAMM_project, LOC_project, LOC_type, LOC_package, LOCNAMM_type, NOMNAMM_package, NOCS_project, Complextype, NOCS_package
FE	09	Method, ATFD_type, Project, AMW_type, package, AMWNAMM_type, complextype, CBO_type, LOC_method
LM	10	Project, Method, CYCLO_method, ATFD_type, complextype, package, LOC_method, NOAV_method, CBO_type, CINT_method

Table 9 shows the result comparison with and without the applied PCA based FST in each dataset with five ML algorithms. We observed the following points for each dataset:

1. For the DC dataset, RF, DT, and KNN models provided higher Severity Accuracy Scores when applied the PCA feature selection technique, while the LR and MLP models achieved better Severity Accuracy Scores without applying PCA. The highest Severity Accuracy Score of 0.83 was achieved by the DT model using the PCA feature selection technique.
2. For the GC dataset, LR, RF, and DT models resulted higher Severity Accuracy Score when applied the PCA feature selection technique. The highest Severity Accuracy Score of 0.85 was achieved by the RF model using the PCA feature selection technique. At the same time, the MLP and KNN models achieved better Severity Accuracy Scores without applying PCA.
3. For the Feature envy dataset, MLP and RF models provided higher Severity Accuracy Scores when applied the PCA feature selection technique, while the LR and KNN models achieved better Severity Accuracy Scores without applying PCA. The highest Severity Accuracy Score of 0.96 was achieved by the MLP and RF model using the PCA feature selection technique. The DT model achieved the same result with and without applied PCA.
4. For the LM dataset, LR, MLP, RF, and DT models resulted higher Severity Accuracy Scores when applied the PCA feature selection technique, while the KNN model achieved a better Severity Accuracy Score without applying PCA. The highest Severity Accuracy Score of 0.99 was achieved by the RF and DT model using the PCA feature selection technique.

**Table 9:** Result comparison between with and without applied PCA based FST.

Dataset	Model name	Severity accuracy score with applied PCA	Severity accuracy score without applied PCA
DC	LR	0.64	**0.66**
	MLP	0.40	**0.42**
	RF	**0.80**	0.76
	DT	**0.83**	0.80
	KNN	**0.58**	0.53
GC	LR	**0.67**	0.66
	MLP	0.43	**0.48**
	RF	**0.85**	0.78
	DT	**0.74**	0.72
	KNN	0.59	**0.61**
FE	LR	0.90	**0.92**
	MLP	**0.96**	0.95
	RF	**0.96**	0.91
	DT	0.94	0.94
	KNN	0.88	**0.90**
LM	LR	**0.94**	0.92
	MLP	**0.96**	0.95
	RF	**0.99**	0.98
	DT	**0.99**	0.98
	KNN	0.96	**0.97**

Significant values are in bold.

## Discussion and result analysis

In this study, three research questions are presented in "Introduction" section. To address the RQ1, we applied five ML algorithms (LR, MLP, RF, DT, and KNN) to the four CSS datasets, and their results are discussed in "Outcomes for data class" to "Outcomes for long method" sections. The achieved results answer RQ1 and found that the RF model is most helpful in detecting the highest Severity Accuracy Score from GC, FE, and LM datasets, and the DT model is most helpful in detecting the highest Severity Accuracy Score from DC and LM datasets.

To address the RQ2, the SMOTE class balancing method is applied to the four CSS datasets discussed in "The impact of SMOTE's Class-balancing method on predictive performance" section. All datasets have four types of severity classes: severity1, severity2, severity3, and severity4, and all classes had a high imbalance among the values. The dataset configuration with severity class is shown in Table 2. Table 7 presents the results of applying SMOTE technique on the CSS datasets with five ML models. The results confirm that most of the models detected the better Severity Accuracy Score for all the datasets when the SMOTE class balancing method is applied.

To address the RQ3, we have applied the PCA technique to the four CSS datasets discussed in "Effect of PCA feature selection technique on the model’s severity accuracy score" section. Table 8 shows the important features selected from each dataset, and Table 9 shows the result comparison between with and without the applied PCA based FST. After comparison, we observed that the PCA is useful for improving the Severity Accuracy Score of all the ML models for all the datasets.

### Evaluation of our results with relevant research studies

This section constructs a comparative summary of proposed approach's result with other relevant research studies. To the best of our knowledge and available literature on CSS detection, only three authors (Fontana et al.^6^; Abdou et al.^12^; Dewangan et al.^25^) have studied the severity dataset. They applied different methodologies, which are shown in Table 10. Table 10 compares our outcomes with Fontana et al.^6^, Abdou et al.^12^, and Dewangan et al.^25^. Fontana et al.^6^ applied eighteen ML models and implemented binary classification, multinomial classification, and regression technique with linear co-relation filter method. Abdou et al.^12^ applied forty binary and multinomial classification techniques with a ranking correlation algorithm. Dewangan et al.^25^ applied seven ML and ensemble methods. Our approach applied five ML models (LR, MLP, RF, DT, and KNN) with PCA-based Feature selection and SMOTE class balancing techniques.

**Table 10:** Evaluation of our findings with relevant research.

Year	Author name	Datasets							
		DC		GC		FE		LM	
		Best algorithm	Severity accuracy score	Best algorithm	Severity accuracy score	Best algorithm	Severity accuracy score	Best algorithm	Severity accuracy score
2017	Fontana et al.^6^	O-Random Forest	0.77	O-Decision Tree	0.74	J48-Pruned	0.93	B-RandomForest	0.92
2022	Abdou et al.^12^	O-R-SMO	0.93	R-B-RF	0.92	R-B-JRIP,O-R-SMO	0.97	R-B-JRIP, O-B-RF, O-R-JRip	0.97
2023	Dewangan et al.^25^	Gradient Boosting	0.88	DT	0.86	DT	0.96	XG Boost	0.99
	Proposed approach	DT	0. 83	RF	0.85	MLP, RF	0.96	RF, DT	0.99

The comparison for each dataset is shown in the following points:

1. For the DC dataset, in our approach, DT model detected the highest Severity Accuracy Score of 0.83, while the Fontana et al.^6^ detected a Severity Accuracy Score of 0.77 applying the O-RF method and Abdou et al.^12^ detected a Severity Accuracy Score of 0.93 applying the O-R-SMO method. Dewangan et al.^25^ detected a Severity Accuracy Score of 0.88 using gradient boosting model. Therefore, the Abdou et al.^12^ approach is good.
2. For the GC dataset, in our approach, the RF model detected the highest Severity Accuracy Score, 0.85, while the Fontana et al.^6^ detected a Severity Accuracy Score of 0.74 by O-DT approach and Abdou et al.^12^ detected a Severity Accuracy Score of 0.92 by R-B-RF approach. Dewangan et al.^25^ detected a Severity Accuracy Score of 0.86 using DT model. Therefore, the Abdou et al.^12^ approach is good.
3. For the FE dataset, in the proposed approach, the MLP and RF model detected the highest Severity Accuracy Score, 0.96, while the Fontana et al.^6^ detected a Severity Accuracy Score of 0.93 applying the J48-Pruned method and Abdou et al.^12^ detected a Severity Accuracy Score of 0.97 applying the R-B-JRIP and O-R-SMO methods. Dewangan et al.^25^ detected a Severity Accuracy Score of 0.96 using DT model. Therefore, the Abdou et al.^12^ approach is good.
4. For the LM dataset, in the proposed approach, the RF and DT model detected the highest Severity Accuracy Score of 0.99, while the Fontana et al.^6^ detected a Severity Accuracy Score of 0.92 applying the B-Random Forest algorithm and Abdou et al.^12^ detected a Severity Accuracy Score of 0.97 applying the R-B-JRIP, O-B-RF, and O-R-JRip algorithms. Dewangan et al.^25^ detected a Severity Accuracy Score of 0.99 using XG boosting model. So, the Dewangan et al.^25^ and our proposed approach is best the LM dataset.

### Comparing machine learning models statistically

From Tables 7 and 9, it is observed that the same types of results are obtained after applying different models to the same dataset. Therefore, the best model out of the two must be chosen in this scenario where two different models produce similar results. To select the best model from the given five ML models, we applied a Paired t-test statistical analysis to see whether there was a statistically substantial distinction between the two ML models, allowing us to use only the best one. N distinct test sets are needed to generate each classifier in this paired t-test. For N test sets, we employed tenfold cross-validation. The statistical analysis was performed to tenfold cross-validation using a Paired t-test. The mean accuracy and standard deviation for each ML model for each dataset were computed in this study.

- *Mean accuracy* For a dataset, a model with a greater mean accuracy performs better than one with a lower mean accuracy.
- *Standard Deviation* A high standard deviation indicates that most of the values in the dataset are spread out over a wide range. And a low standard deviation indicates that most of the values in the dataset are close to the mean. As a result, the model with the lowest standard deviation is the best choice.

We used tenfold cross-validation and a significance value of 0.05 to calculate the statistical analysis. Table 11 shows the mean accuracy and standard deviation of each classification model across each code-smell dataset. Table 11 shows that the LR model had a 0.01 standard deviation and 0.99 mean accuracy scores for the DC dataset. The LR model achieved the highest 1.00 mean accuracy score for the GC and LM datasets with a 0.00 standard deviation. Additionally, the LR model had a 0.02 standard deviation and a highest mean accuracy score of 0.97 for the FE dataset. As a result, the LR model is determined to be the best model for the severity detection of the four code smell datasets because it has a high mean accuracy and a low standard deviation across all datasets.

**Table 11:** Statistical analysis.

ML models	Mean accuracy for DC	Standard deviation for DC	Mean accuracy for GC	Standard deviation for GC	Mean accuracy for FE	Standard deviation for FE	Mean accuracy for LM	Standard deviation for LM
LR	0.99	0.01	1.00	0.00	0.97	0.02	1.00	0.00
MLP	0.79	0.17	0.77	0.28	0.82	0.01	0.77	0.28
RF	0.98	0.02	0.98	0.02	0.93	0.02	0.98	0.02
DT	0.98	0.02	0.97	0.02	0.92	0.02	0.96	0.02
KNN	0.82	0.06	0.95	0.01	0.89	0.01	0.95	0.01

## Conclusion

Class imbalance issues are significant primary challenges in the CSS dataset. We have considered four CSS datasets: GC, DC, LM, and FE. Five ML models were applied over four CSS datasets. SMOTE method was applied to avoid the class imbalance problem. We also compared performances without using SMOTE techniques. We have also applied the PCA-based FST technique and compared performances without using PCA techniques. The conclusions, obtained from study are presented below.

1. From the Data class dataset highest Severity Accuracy Score of 0.83 was detected by the DT model using eight features selected by the PCA feature selection technique.
2. From the God class dataset highest Severity Accuracy Score of 0.85 was detected by the RF model using nine features selected by the PCA feature selection technique.
3. From the Feature envy dataset highest Severity Accuracy Score of 0.96 was detected by the MLP and RF model using nine features selected by the PCA feature selection technique.
4. From the Long Method dataset highest Severity Accuracy Score of 0.99 was detected by the RF and DT model using ten features selected by the PCA feature selection technique.

Ensemble learning has a good scope to be applied in the CSS dataset. Deep learning-based models are still not possible because of the small number of instances in a dataset; however, by using data augmentation, we may increase the size of our training set so that deep learning-based models can be effectively applied. The deep learning methods and other FST techniques can be used in future studies.

## Data Availability

All these datasets are accessible at http://essere.disco.unimib.it/reverse/MLCSD.html, Fontana et al.^6^.

## Appendix

See Table 12.

**Table 12:** Selected metrics description^6^.

Quality measure	Selected metric	Metric name	Granularity
Size	LOC_project	Lines of code	Project, class, package
	LOC_package		
	LOC_type		
	NOMNAMM_project	Number of not accessor or mutator methods	Project, class, package
	NOMNAMM_package		
	LOCNAMM_type	Lines of code without accessor or mutator methods	Class
	NOCS_project	Number of classes	Project, package
	NOCS_package		
–	Complextype	–	Class
–	Method	–	–
Complexity	ATFD_type	Access to foreign data	Method
–	Project	–	–
Size	AMW_type	Average methods weight	Class
–	Package	–	–
Complexity	AMWNAMM_type	Average methods weight of not accessor or mutator methods	Class
–	complextype	–	Method
Coupling	CBO_type	Coupling between objects classes	Class
Size	LOC_method	Lines of code	Method
	CYCLO_method	Cyclomatic complexity	
Complexity	NOAV_method	Number of accessed variables	
Coupling	CINT_method	Coupling intensity	
